# Challenges and opportunities in the creation and implementation of cancer-control plans in Africa

**DOI:** 10.3332/ecancer.2019.938

**Published:** 2019-07-25

**Authors:** Kalina Duncan, Mishka Kohli Cira, Prebo Barango, Edward Lloyd Trimble

**Affiliations:** 1 National Cancer Institute Center for Global Health, National Cancer Institute, 9609 Medical Center Drive, Rockville, MD 20850, USA; 2 Intercountry Support Team East and Southern Africa, World Health Organization Africa Regional Office, 86 Enterprise Road, Highlands, Harare, Zimbabwe

**Keywords:** cancer control, Africa, NCCP, implementation

## Abstract

Cancer on the African continent is quickly becoming an overt public health crisis due to an aging population and changes in lifestyle. The World Health Organization (WHO) states that a national cancer-control programme should aim to reduce cancer incidence and mortality and improve quality of life of cancer patients, through a national cancer-control plan (NCCP) that is systematic, equitable and evidence-based. Despite this, only 11 countries in Africa have a current NCCP. Participants in a US National Cancer Institute-supported, multi-year, technical assistance programme for cancer-control planning noted three main opportunities to improve how plans are created and implemented: 1) mobilisation of resources and partners for plan implementation; 2) accurate surveillance data to promote better resourcing of NCCPs; and, 3) sustainable and innovative partnership models to strengthen capacity to implement NCCPs. Most countries in the region face similar challenges in the development and implementation of an NCCP, including inadequate human, technical, and financial resources. Collaborative partnerships increase access to evidence-based cancer-control planning tools, mentoring and technical assistance, and have the potential to bridge the capacity gap and catalyse better implementation of NCCPs. Challenges can be overcome by better leveraging these opportunities to address the gaps that inhibit cancer control in Africa.

## Introduction

Cancer on the African continent is quickly becoming an overt public health crisis due to an aging population and changes in lifestyle, including increased tobacco and alcohol use, rising obesity and physical inactivity. Unchecked, the burden of cancer in low- and middle-income countries will increase by more than 60% by 2030 [1], plaguing already overburdened health systems. In addition, 19 of the 20 countries with the highest incidence of cervical cancer worldwide are in Africa [2].

The World Health Organization (WHO) states that a national cancer-control programme should aim to reduce cancer incidence and mortality and improve quality of life of cancer patients, through a national cancer-control plan (NCCP) that is systematic, equitable and evidence-based [3]. A comprehensive and realistic NCCP serves as a centralised resource for stakeholders to understand and align activities with a country’s priority areas, and to generate resources for plan implementation [4]. In 2008, when few countries in Africa had NCCPs [5], the WHO African Regional Office adopted the first-ever regional strategy for the prevention and control of cancer in Africa [6]. Five years later, the African Organisation for Research and Training in Cancer (AORTIC) released the Cancer Plan for the African Continent 2013–2017, that addressed overarching goals and strategies for priority cancers in the region [7]. Furthermore, the World Health Assembly has passed resolutions that call for the development and implementation of costed, comprehensive and integrated NCCPs [8].

Even with multiple calls for strengthened national cancer-control planning measures, a 2018 analysis of the International Cancer Control Partnership (ICCP) portal shows that a total of only 11 countries (20.1%) in the Africa region have a current NCCP [9]. Given the priority of reducing mortality from cancer and other non-communicable diseases previously stated, more coverage and resourced implementation of costed NCCPs is necessary to achieve the goals laid out in the WHO, AORTIC and UN plans and declarations.

## Background and methods

Building on the US government’s domestic engagement in comprehensive cancer-control planning [10], the US National Cancer Institute (NCI), Center for Global Health (CGH) launched the International Cancer Control Leadership Forum (ICCLF) Program in 2013. The ICCLF convened multi-stakeholder country teams from multiple regions for technical training and assistance on cancer-control principles and strategies. CGH held a sub-Saharan African ICCLF in 2014 in Lusaka, Zambia, followed by a virtual sub-Saharan African ICCLF in 2017, with attendees from 9 to 10 countries, respectively, plus multiple partner institutions. In 2018, to continue this community of practice, CGH initiated the tele-mentoring Africa Cancer Research and Control Project ECHO® (Extension for Community Healthcare Outcomes) Program.

**Figure 1.**
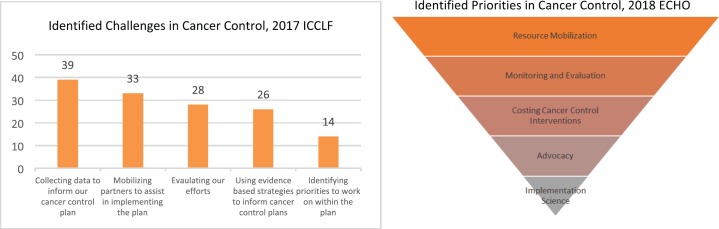
Challenges and priorities in national cancer-control planning identified by 2017 ICCLF and 2018 ECHO participants.

In order to understand the challenges and priorities in cancer-control planning for curriculum development purposes, CGH administered baseline surveys to participants of the 2017 ICCLF (response rate 73%) and 2018 ECHO (response rate 77%), prior to each programme's launch, via Google Forms. The most-often identified challenges and priorities can be seen in Figure 1 [11] and were referenced to frame the commentary on the challenges and opportunities presented below.

## Challenges and opportunities in cancer-control planning in Africa: mobilising resources, creating and leveraging partnerships and utilizing reliable data for cancer-control plan development and implementation

### Mobilisation of resources and partners for plan implementation

Participants identified resource and partner mobilisation for NCCP implementation as two of the leading challenges they face (Figure 1). Despite the 2001 Abuja Declaration, whereby countries in the Africa region committed to spend at least 15% of the government budget on public health, only four countries in the region met that target by 2014 [12]. Cancer-control planners are faced with implementing cancer-control policy with suboptimal budget allocation needed to secure the necessary human, technical, educational and infrastructure resources.

An additional barrier is the organisation of national cancer programmes within ministries of health (MoH). In most African countries, the structure is inconsistent, creating an obstacle to mobilise resources and partners for NCCP implementation. National cancer programmes are, at times, part of the NCD programme under the coordination of a cancer-control officer or an NCD programme officer (the latter often equates to no dedicated cancer-control budget line in the national budget), a standalone cancer-control programme, or one divided across MoH units (with prevention and clinical management of cancer handled by different units/departments within the MoH structure).

Unlike the global HIV/AIDS response, a centralised pool of financial and technical resources does not exist for cancer and other NCDs. Therefore, MoH technical officers must mobilise resources and engage non-governmental implementing partners as a crucial step for successful NCCP implementation. The suboptimal level of coordination of cancer prevention and control within most ministries of health in Africa means that agreement on priorities, which is necessary for partner and stakeholder engagement, is less efficient and thus, impacts funding for the NCCP.

Opportunities to make progress in this critical area are emerging. The increasing number of health economists now working on NCDs can support governments in assessing the cost (both financial and otherwise) and affordability of interventions to prevent and control cancer and other NCDs [13]. The WHO is adapting the OneHealth Tool, a planning resource for measuring both financial sustainability and level of health impact across disease groups and health systems [14], to incorporate key cancer and NCD-related interventions. The Ethiopian Federal MoH was able to utilise the costing principles outlined in the existing OneHealth tool to fully cost the National Cancer Control Plan, 2016–2020 [15], demonstrating that use of existing tools and on-the-ground expertise can overcome perceived barriers.

### Accurate surveillance data to promote better resourcing of NCCPs

The collection of data to inform NCCP efforts was the top challenge identified by 2017 ICCLF participants (Figure 1). Robust and sustainable population-based cancer registries are crucial for measuring the burden of cancer in a community, assessing the impact of interventions, and conducting critical etiological research [16]. They also provide relevant data to ensure that an NCCP is accurately prioritised, costed, operationalised and evaluated [17]. And yet, in the most recent volume of *Cancer Incidence in Five Continents* (Volume XI), only seven population-based cancer registries from six African countries had sufficient data quality and capture [18] for inclusion.

Governments across Africa have an opportunity to prioritise and resource population-based cancer registries to enable ministries of health to develop, cost, resource, implement and evaluate NCCPs in a way that truly benefits populations. Nigeria is an example of where sustainable and long-standing cancer registries, coordinated under the Federal MoH, have provided national estimates of the increasing cancer burden which allowed decision-makers to subsequently inform policies and most recently, the National Cancer Control Strategy (2018–2022). The ability to prioritise which cancers to address will allow for strategic partner engagement toward effective implementation [19].

### Sustainable and innovative partnership models to strengthen capacity to implement NCCPs

A primary challenge for cancer-control planning in Africa, and perhaps the most commonly identified in the literature, is the lack of essential health system capacity and infrastructure needed to prevent, detect and treat cancer [20, 21]. Human resource shortages, or so-called “brain drain” of medical practitioners, and a lack of basic medical support staff contribute to generally poor outcomes in cancer control. Better coordination of care and more resources and skilled professionals at primary and secondary health facilities would lessen the burden on tertiary health centres and help ensure more equitable implementation of NCCPs [20].

There is growing momentum among medical schools and universities in Africa to develop specialised oncology-related diploma courses and fellowships [22, 23]. While this is a positive development, the impact of these training programmes will take time to be reflected in the cancer workforce and improved timeliness and outcomes of cancer treatment. In the meantime, and to complement these training programmes, there are encouraging knowledge-transfer solutions through technology and partnership. For example, the Project ECHO® tele-mentoring model was developed by the University of New Mexico and utilises low-cost technology to promote knowledge sharing and network building through a virtual platform. The acceptability and feasibility of such exchanges, in combination with traditional learning platforms, have the potential to help improve knowledge and eventually improve the lives of underserved populations struggling with cancer [24–26].

Cancer-care-focused partnerships such as the Academic Model Providing Access to Healthcare in Eldoret, Kenya, Partners in Health in Butaro, Rwanda, and the Fred Hutchinson Cancer Research Center Partnership with the Uganda Cancer Institute, show how a long-standing commitment to collaboration and partnership [27, 28] can sustainably increase cancer-control capacity. These partnerships have been shown to increase not only clinical research and treatment capabilities, but also to strengthen pathology, cancer registries, awareness, national health systems and health policies [29].

Finally, while this paper is focused on challenges specific to cancer-control in Africa, there are lessons to be learnt globally from similar challenges and solutions in other regions, where building research networks with a focus on implementation science questions to inform effective programming have propelled the effective implementation of national cancer-control plans [30]. Additionally, the aforementioned ICCP fosters opportunities for countries to identify potential partners for NCCP development and implementation, operationalisation of the WHO OneHealth and other costing tools, and development of trans-regional communities of practice, such as those listed above. A recent *Lancet Oncology* analysis of national cancer-control plans, led by the ICCP, WHO, and Union for International Cancer Control, reveals that the number of plans worldwide has increased and the focus is now on successful plan implementation. The authors cite examples from Africa where planning has led to action, both in cervical cancer screening and in population-based cancer registries [31]. These examples demonstrate that the progress made in different regions is generalisable for improved cancer control worldwide.

## Conclusion

Reduction in mortality and morbidity from cancer are keys to the attainment of the WHO voluntary global target of 25% reduction in premature mortality from NCDs by 2025 as well as the Sustainable Development Goal target of a one-third reduction in premature mortality from NCDs by 2030. In order to achieve cancer-related global targets, a well-organised and resourced NCCP, guided by accurate data on the burden of cancer and related risk factors, is necessary. Most countries in the region face similar challenges in the development and implementation of an NCCP, including inadequate human, technical and financial resources. Collaborative partnerships increase access to evidence-based cancer-control planning tools, mentoring and technical assistance, and have the potential to bridge the capacity gap and catalyse better implementation of NCCPs. There is tremendous opportunity in the continued support of these kinds of partnerships for cancer-control programmes in Africa.

## Conflicts of interest

The authors have no conflicts of interest to disclose.

## Funding

This project has been funded in whole or in part with US federal funds from the US National Cancer Institute, National Institutes of Health, and under Contract No. HHSN261200800001E. The content of this publication does not necessarily reflect the views or policies of the US Department of Health and Human Services or National Institutes of Health, nor does mention of trade names, commercial products, or organisations imply endorsement by the US government.

## Contributors

KD wrote or co-wrote the introduction, background and methods, challenges, and opportunities sections, edited and revised the paper. MKC co-wrote the background and methods, challenges, opportunities, edited and revised the paper. PB wrote or co-wrote the conclusion and background, edited, and revised the paper. ELT edited and revised the paper.
